# Exposure to residential green and blue space and the natural environment is associated with a lower incidence of psychiatric disorders in middle-aged and older adults: findings from the UK Biobank

**DOI:** 10.1186/s12916-023-03239-1

**Published:** 2024-01-15

**Authors:** Bao-Peng Liu, Rachel R. Huxley, Tamara Schikowski, Ke-Jia Hu, Qi Zhao, Cun-Xian Jia

**Affiliations:** 1 Department of Epidemiology, School of Public Health, Cheeloo College of Medicine, Shandong University, Jinan, 250012 Shandong China; 2 Faculty of Health, Deakin University, Melbourne, VIC 3000 Australia; 3 Department of Epidemiology, IUF-Leibniz Research Institute for Environmental Medicine, Düsseldorf, Germany; 4 Department of Big Data in Health Science, School of Public Health, Zhejiang University, Zijingang Campus, Hangzhou, 310058 China; 5 Shandong University Climate Change and Health Center, Shandong University, Jinan, 250012 Shandong China

**Keywords:** Green space, Blue space, Natural environment, Psychiatric disorder, UK Biobank

## Abstract

**Background:**

There is increasing evidence for the role of environmental factors and exposure to the natural environment on a wide range of health outcomes. Whether exposure to green space, blue space, and the natural environment (GBN) is associated with risk of psychiatric disorders in middle-aged and older adults has not been prospectively examined.

**Methods:**

Longitudinal data from the UK biobank was used. At the study baseline (2006–2010), 363,047 participants (women: 53.4%; mean age 56.7 ± 8.1 years) who had not been previously diagnosed with any psychiatric disorder were included. Follow-up was achieved by collecting records from hospitals and death registers. Measurements of green and blue space modeled from land use data and natural environment from Land Cover Map were assigned to the residential address for each participant. Cox proportional hazard models with adjustment for potential confounders were used to explore the longitudinal associations between GBN and any psychiatric disorder and then by specific psychiatric disorders (dementia, substance abuse, psychotic disorder, depression, and anxiety) in middle-aged and older adults.

**Results:**

During an average follow-up of 11.5 ± 2.8 years, 49,865 individuals were diagnosed with psychiatric disorders. Compared with the first tertile (lowest) of exposure, blue space at 300 m buffer [hazard ratio (HR): 0.973, 95% confidence interval (CI): 0.952–0.994] and natural environment at 300 m buffer (HR: 0.970, 95% CI: 0.948–0.992) and at 1000 m buffer (HR: 0.975, 95% CI: 0.952–0.999) in the third tertile (highest) were significantly associated with lower risk of incident psychiatric disorders, respectively. The risk of incident dementia was statistically decreased when exposed to the third tertile (highest) of green space and natural environment at 1000 m buffer. The third tertile (highest) of green space at 300 m and 1000 m buffer and natural environment at 300 m and 1000 m buffer was associated with a reduction of 30.0%, 31.8%, 21.7%, and 30.3% in the risk of developing a psychotic disorder, respectively. Subgroup analysis suggested that the elderly, men, and those living with some comorbid conditions may derive greater benefits associated with exposure to GBN.

**Conclusions:**

This study suggests that GBN has significant benefits for lowering the risk of psychiatric disorders in middle-aged and older adults. Future studies are warranted to validate these findings and to understand the potential mechanistic pathways underpinning these novel findings.

**Supplementary Information:**

The online version contains supplementary material available at 10.1186/s12916-023-03239-1.

## Background

Psychiatric disorders comprise a wide number of conditions and rank among the most important contributors to the global burden of disease. Depressive disorder, anxiety disorder, and schizophrenia are the top three specific psychiatric disorders in terms of disability-adjusted life-years (DALYs) [1]. In 2019, the estimated age-standardized prevalence for any psychiatric disorder in men was 11.7 cases per 100 individuals and 12.8 in women [1]. Women also have a higher age-standardized DALY rate of psychiatric disorders compared with men (1.4 vs. 1.7 per 100 individuals). The ranked leading cause of DALYs associated with psychiatric disorders has steadily increased from 13 in 1900 to 7 in 2019 [1]. Receiving a diagnosis of having a psychiatric condition is associated with a greater propensity towards suicide and self-harm [2].

In recent years, there has been increasing awareness of the impact of the environment on an individuals’ health. Green space, blue space, and the natural environment typically refer to open space for greening or leisure, rivers, lakes, or seas, and the residential non-building space, respectively. There is accumulating evidence from cross-sectional and prospective studies that exposure to green space, blue space, and the natural environment (GBN) has beneficial effects on health, especially for individuals living with certain chronic diseases such as cardiorespiratory diseases [3], type 2 diabetes [4], chronic kidney diseases [5], and inflammatory bowel diseases [6]. By contrast, the association between GBN and psychiatric disorders remains less well defined. Previous studies have reported a protective effect of green space on risk of dementia [7, 8], depression [9–13], anxiety [11, 14], and other mental issues [15], while other studies reported null associations [16–18]. Inconsistent results have also been shown for the associations of blue space with depression [12, 13, 18] and anxiety [12, 14, 19], respectively. In addition, a series of meta-analyses suggested that exposure to the natural environment could decrease the risk of depression [20] and anxiety [21]. However, few studies have examined the effect of GBN on specific psychiatric disorders, especially psychotic disorders. Moreover, most aforementioned studies have been cross-sectional and have only been able to explore the association between a single GBN component and risk of certain psychiatric disorders. To date, there is limited prospective evidence to examine the relationship between exposure to GBN with incident psychiatric events.

In the current study, we aimed to explore the association between exposure to residential GBN with any or specific psychiatric disorders among middle-aged and older adults in the UK Biobank (UKB), a prospective cohort study of more than half a million adults. We also sought to examine whether there are subgroups of the population who might derive particular benefit from exposure to GBN.

## Methods

### Study design and participants

Data was derived from UKB, which is an ongoing prospective cohort study [22]. Initially, more than 500,000 participants (aged 37–73 years) were recruited during baseline (2006–2010) from 22 research centers across the UK (England, Wales and Scotland). More details about the locations are available at https://biobank.ndph.ox.ac.uk/showcase/exinfo.cgi?src=UKB_centres_map. After obtaining electronic consent for the use of de-identified data, every participant completed a self-completed touch-screen questionnaire, a computer-assisted interview. Participants also consented to a range of physical measures as well as sampling assays and genotyping [22]. For this study, we excluded participants with a recorded history at baseline (based on the date of diagnosis) of any psychiatric disorder as well as those individuals with missing data for GBN at study baseline. Follow-up of health-related outcomes was achieved by matching any record from the national health-related hospitals, primary care, death registers, and other systems. A total of 363,047 participants were included in the analysis (Additional file 1: Fig. S1).

## Measures

### Exposures

With consideration of existing evidence of the associations between GBN density and health outcomes and relevant public policy [23–25], the percentage of GBN assigned to the 300 m and 1000 m buffers for each residential location [6, 26] was used to estimate an individual’s combined GBN exposure (which took into account their residential location as well as the GBN in the wider-area relative to the residential location) [27, 28]. The percentage of residential green space and blue space, which were classed as “greenspace” and “water,” were proportions of the total percentage of all land-use types. In line with previous UKB studies exploring the health effect of GBN [29, 30], data on green and blue space were collected from the 2005 Generalized Land Use Database (GLUD) for England [26]. GLUD, which was obtained from Neighborhood Statistics (http://www.neighbourhood.statistics.gov.uk/), provided data on land use distribution for 2001 Census Output Areas in England. The data on the distribution of natural environment were collected from Land Cover Map (LCM) 2007 (25 m*25 m) [31]. The LCM 2007 product was from Centre for Ecology and Hydrology (CEH) [32] and included 23 land cover classes with Class 1–21 reclassified as natural environment. Notedly, Class 22–23 included buildings and gardens, which were different from the definition of GLUD measure. The definition of natural environment was partially overlapped with green and blue space in this study. Participants out of England were excluded due to the restricted availability of GLUD data. More details could be seen at the website of UKB: https://biobank.ndph.ox.ac.uk/showcase/field.cgi?id=24507.

### Outcomes

The diagnoses of any or specific psychiatric disorder were obtained from the “first occurrence fields” provided by UKB (data category: 2409), which included data from primary care, hospital inpatient record, self-reported medical conditions, and death registers [22, 33]. Any psychiatric disorder (F00-F99) was coded using the International Classification of Disease, 10th version (ICD-10) [34]. Considering the higher prevalence rate in the general population, this study also examined certain specific psychiatric disorders, including dementia (F00-F03), substance abuse (F10-F19), psychotic disorder (F20-F29), depression (F32-F33), and anxiety (F40-F41) [1, 34, 35]. More details about the outcomes are provided in Additional file 1: Table S1. Participants entered the cohort from the date of being recruited and exited at the date of death, occurrence of outcomes, or censorship, whichever came first. The date of censorship was derived from the date of diagnosis of a psychiatric disorder, obtained from the section of first occurrence field.

### Covariates

The covariates in this study were selected by reviewing previous studies related to psychiatric disorders [36, 37], including age, sex, ethnicity, socioeconomic status (SES), body mass index (BMI), household income before tax per year, education group, smoking status, alcohol drinker status, physical activity, history of hypertension, and type 2 diabetes (T2D). The category of physical activity (data field of UKB: 22,032) was derived from the Metabolic Equivalent Task (MET) score that was based on the International Physical Activity Questionnaire (IPAQ) guidelines [38]. SES was measured by Townsend area deprivation index [39]. A higher score indicated greater socioeconomic deprivation and poor SES and quartiles of the score for SES were included in the analyses. The diagnoses of hypertension and T2D were also obtained from first occurrence fields. The data field and definition of other covariates are shown in Additional file 1: Table S2.

### Statistical analysis

Multivariate imputations by chained equations (MICE) [40] were used to impute missing values with a proportion lower than 5%. The missing values for income (15.8%) and physical activity (19.7%) were regarded as a classification in the models, respectively. Initially, two series of Cox proportional hazard models were performed to explore the associations of GBN and all or specific psychiatric disorders, respectively. Model 1 was adjusted for age and sex, and model 2 was additionally adjusted for ethnicity, SES, BMI, household income before tax per year, education group, smoking status, alcohol drinker status, physical activity, hypertension, and T2D. To take into account the potential for collinearity, measures of GBN were included in separately adjusted models. Tertiles of exposures were used as cut-offs, with the first tertile (the lowest) set as the reference group. An ordinal scale based on the tertiles was also used to explore the continuous trend of the exposure to psychiatric disorders. There were no obvious violations to the proportional hazards assumption for interested exposures.

Stratified analyses by age (less than 65 years vs. 65 years or above), sex (female vs. male), ethnicity (white vs. non-white), SES (good, the first two quantiles vs. poor, the second two quantiles), BMI (normal or underweight vs. overweight), income (low, < £52,000 vs high, ≥ £52,000), education (college or university vs. others), smoking status (never vs. previous/current), alcohol drinking (less than once per week vs. once per week or above), physical activity (high vs. low or moderate), history of hypertension, and T2D at baseline were performed to observe the different effects of the exposures of interest on incident psychiatric disorders. *Z* test was used to compare the estimates of different subgroups as recommended by Altman et al. [41].

We performed several sensitivity analyses to verify the robustness of the main findings. First, we omitted the participants with any psychiatric disorder during the first 2 years of follow-up to account for reverse causality. Second, the participants with missing values of the covariates were excluded from the analyses (< 5% of participants). Third, considering the interactive effect of the exposure and air or noise pollution on psychiatric disorders, we separately added air pollution [particular matter 10 (PM_10_)] and noise pollution (annual average of 24 h noise) to the models. PM_10_ and noise pollution were estimated by LUR model developed as part of the European Study of Cohorts for Air Pollution Effects (ESCAPE, http://www.escapeproject.eu/). Fourth, outdoor time in winter and autumn and a history of consultation with a psychiatrist or general practitioner (GP) were further adjusted for in the models to explore the relative effects on the associations, respectively. Finally, a range of percentile classifications for GBN exposure were performed to test for the robustness of the findings: ($\le$ 50 and > 50 percentile); ($\le$ 20, > 20 to 80, and > 80 percentile), and four groups ($\le$ 25,  > 25 to 50, > 50 to 75, and > 75 percentile).

All the statistical analyses were performed by R v4.1.2 software, with package “mice” used for imputation.

## Results

At baseline, 53.4% of participants identified as women and the mean age of all participants was 56.7 (± 8.1 years; Table 1). During the average follow-up of 11.5 (± 2.8) years, the incidence rate for any psychiatric disorder was 11.48 per 1000 person-years in women and 12.45 in men. Individuals diagnosed with any psychiatric disorder were more likely to be men, have chronic health conditions and exhibit suboptimal lifestyle behaviors, and have a lower SES compared with other groups in the cohort.

**Table 1:** The descriptive statistics by any psychiatric disorder

Characteristics*	Overall (*n* = 363,047)	Any psychiatric disorder		*P for differences*
		**No (*n* = 313,182)**	**Yes (*n* = 49,865)**	
Sex, *n* (%)				< 0.001
Female	193,854 (53.4)	168,043 (53.7)	25,811 (51.8)	
Male	169,193 (46.6)	145,139 (46.3)	24,054 (48.2)	
Age, mean (SD)	56.70 (8.12)	56.55 (8.08)	57.64 (8.29)	< 0.001
Ethnicity, *n* (%)				< 0.001
White	338,938 (93.4)	292,197 (93.3)	46,741 (93.7)	
Others	22,056 (6.1)	19,305 (6.2)	2751 (5.5)	
Unknown	2053 (0.6)	1680 (0.5)	373 (0.7)	
Socioeconomic status, *n* (%) #				< 0.001
First quartile	90,706 (25.0)	80,549 (25.7)	10,157 (20.4)	
Second quartile	90,645 (25.0)	79,724 (25.5)	10,921 (21.9)	
Third quartile	90,671 (25.0)	78,468 (25.1)	12,203 (24.5)	
Fourth quartile	90,674 (25.0)	74,139 (23.7)	16,535 (33.2)	
Unknown	351 (0.1)	302 (0.1)	49 (0.1)	
BMI, *n* (%)				< 0.001
Underweight or normal weight (< 24.9 kg/m^2^)	121,840 (33.6)	107,052 (34.2)	14,788 (29.7)	
Overweight (25.0–29.9 kg/m^2^)	154,670 (42.6)	134,072 (42.8)	20,598 (41.3)	
Obese ($\ge$ 30 kg/m^2^)	84,384 (23.2)	70,414 (22.5)	13,970 (28.0)	
Unknown	2153 (0.6)	1644 (0.5)	509 (1.0)	
Household income before tax per year (£), *n* (%)				< 0.001
Less than 18,000	63,916 (17.6)	51,157 (16.3)	12,759 (25.6)	
18,000–30,999	77,560 (21.4)	66,461 (21.2)	11,099 (22.3)	
31,000–51,999	80,871 (22.3)	71,472 (22.8)	9399 (18.8)	
52,000–100,000	65,326 (18.0)	59,235 (18.9)	6091 (12.2)	
Greater than 100,000	18,140 (5.0)	16,930 (5.4)	1210 (2.4)	
Unknown	57,234 (15.8)	47,927 (15.3)	9307 (18.7)	
Education group, *n* (%)				< 0.001
College or university degree	117,426 (32.3)	105,511 (33.7)	11,915 (23.9)	
Any school degree (A-level, AS-level, O-level, GCSE, CSE)	137,056 (37.8)	118,757 (37.9)	18,299 (36.7)	
Vocational qualification (NVQ, HND, or HNC) or other professional qualifications	42,031 (11.6)	35,715 (11.4)	6316 (12.7)	
None of the above	58,382 (16.1)	46,696 (14.9)	11,686 (23.4)	
Unknown	8152 (2.2)	6503 (2.1)	1649 (3.3)	
Smoking status, *n* (%)				< 0.001
Never	207,476 (57.1)	187,640 (59.9)	19,836 (39.8)	
Previous	124,768 (34.4)	107,671 (34.4)	17,097 (34.3)	
Current	28,623 (7.9)	16,137 (5.2)	12,486 (25.0)	
Unknown	2180 (0.6)	1734 (0.6)	446 (0.9)	
Alcohol drinker status, *n* (%)				< 0.001
Daily or almost daily	74,429 (20.5)	63,106 (20.1)	11,323 (22.7)	
Above one time per week	180,021 (49.6)	158,186 (50.5)	21,835 (43.8)	
One to three times a month	39,780 (11.0)	34,344 (11.0)	5436 (10.9)	
Special occasions only	40,442 (11.1)	33,982 (10.9)	6460 (13.0)	
Never	27,334 (7.5)	22,760 (7.3)	4574 (9.2)	
Unknown	1041 (0.3)	804 (0.3)	237 (0.5)	
Total physical activity, *n* (%)				< 0.001
Low	52,863 (14.6)	44,917 (14.3)	7946 (15.9)	
Moderate	118,932 (32.8)	103,994 (33.2)	14,938 (30.0)	
High	119,726 (33.0)	104,452 (33.4)	15,274 (30.6)	
Unknown	71,526 (19.7)	59,819 (19.1)	11,707 (23.5)	
Hypertension				< 0.001
No	268,766 (74.0)	235,153 (75.1)	33,613 (67.4)	
Yes	94,281 (26.0)	78,029 (24.9)	16,252 (32.6)	
Type 2 diabetes				< 0.001
No	345,223 (95.1)	299,239 (95.5)	45,984 (92.2)	
Yes	17,824 (4.9)	13,943 (4.5)	3881 (7.8)	

*SD* Standard deviation, *BMI* Body mass index

^*^Unknown included prefer not to answer, do not know, and missing value in the database of UK biobank

^#^Socioeconomic status was measured by Townsend area deprivation index

As shown in Table 2, there were positive effects for the associations of blue space (the third tertile) [300 m buffer, HR:0.973, 95% CI: 0.952–0.994)] and natural environment (the third tertile) [300 m buffer, HR:0.970, 95% CI: 0.948–0.992); 1000 m buffer, HR:0.975, 95% CI: 0.952–0.999)] with any psychiatric disorder. Similar associations were also found when including an ordinal scale in the respective models. However, no statistically significant associations were observed between green space at 300 m or 1000 m buffer and blue space at 1000 m buffer (the third tertile) with any psychiatric disorder.

**Table 2:** The independent associations of green, blue space and natural environment with any psychiatric disorder (*n* = 363,047)

Exposure	Person-years (PYs)	Psychiatric disorder (*n*)	Incidence rate, (*n*/1000 PYs, 95% CI)	Model 1, HR (95% CI)	Model 2, HR (95% CI)
**Overall**	4,180,295.3	49,865	11.93 (11.82–12.03)		
**Green space, 300 m buffer**					
First tertile (the lowest)	1,378,805.6	17,062	12.37 (12.19–12.56)	Reference	Reference
Second tertile	1,391,679.4	17,568	12.62 (12.44–12.81)	1.009 (0.988–1.031)	1.010 (0.988–1.031)
Third tertile (the highest)	1,409,810.3	15,235	10.81 (10.64–10.98)	0.856 (0.837–0.875) ***	0.984 (0.961–1.006)
Ordinal scale				0.927 (0.917–0.937) ***	0.992 (0.981–1.004)
**Blue space, 300 m buffer**					
First tertile (the lowest)	1,379,691.0	17,326	12.56 (12.37–12.75)	Reference	Reference
Second tertile	1,397,693.1	16,310	11.67 (11.49–11.85)	0.920 (0.901–0.940) ***	0.983 (0.961–1.004)
Third tertile (the highest)	1,402,911.2	16,229	11.57 (11.39–11.75)	0.913 (0.893–0.932) ***	0.973 (0.952–0.994) *
Ordinal scale				0.955 (0.945–0.965) ***	0.986 (0.976–0.997) *
**Natural environment, 300 m buffer**					
First tertile (the lowest)	1,375,925.1	17,640	12.82 (12.63–13.01)	Reference	Reference
Second tertile	1,402,678.0	17,160	12.23 (12.05–12.42)	0.941 (0.921–0.961) ***	1.003 (0.982–1.025)
Third tertile (the highest)	1,401,692.2	15,065	10.75 (10.58–10.92)	0.818 (0.801–0.836) ***	0.970 (0.948–0.992) **
Ordinal scale				0.905 (0.896–0.915) ***	0.985 (0.974–0.997) **
**Green space, 1000 m buffer**					
First tertile (the lowest)	1,371,142.3	17,556	12.80 (12.62–12.99)	Reference	Reference
Second tertile	1,396,052.3	17,368	12.44 (12.26–12.63)	0.951 (0.932–0.972) ***	1.013 (0.991–1.035)
Third tertile (the highest)	1,413,100.7	14,941	10.57 (10.41–10.74)	0.802 (0.785–0.820) ***	0.983 (0.960–1.007)
Ordinal scale				0.897 (0.888–0.907) ***	0.992 (0.980–1.004)
**Blue space, 1000 m buffer**					
First tertile (the lowest)	1,387,287.6	16,938	12.21 (12.03–12.39)	Reference	Reference
Second tertile	1,394,469.2	16,183	11.61 (11.43–11.79)	0.947 (0.927–0.968) ***	0.974 (0.953–0.995) *
Third tertile (the highest)	1,398,538.5	16,744	11.97 (11.79–12.16)	0.977 (0.957–0.998) *	0.981 (0.960–1.002)
Ordinal scale				0.988 (0.978–0.999) *	0.990 (0.980–1.001)
**Natural environment, 1000 m buffer**					
First tertile (the lowest)	1,370,786.7	18,053	13.17 (12.98–13.36)	Reference	Reference
Second tertile	1,397,814.0	17,070	12.21 (12.03–12.40)	0.906 (0.887–0.925) ***	1.014 (0.992–1.036)
Third tertile (the highest)	1,411,694.5	14,742	10.44 (10.28–10.61)	0.769 (0.752–0.786) ***	0.975 (0.952–0.999) *
Ordinal scale				0.878 (0.868–0.887) ***	0.988 (0.976–1.000) *

*HR* Hazard ratio, *CI* Confidence interval

^*^P < 0.05, **P < 0.01, ***P < 0.001

Model 1 was adjusted for age and sex. Model 2 was further adjusted for ethnicity, socioeconomic status, BMI, household income, education group, smoking status, alcohol drinker status, and physical activity

**Fig. 1:**
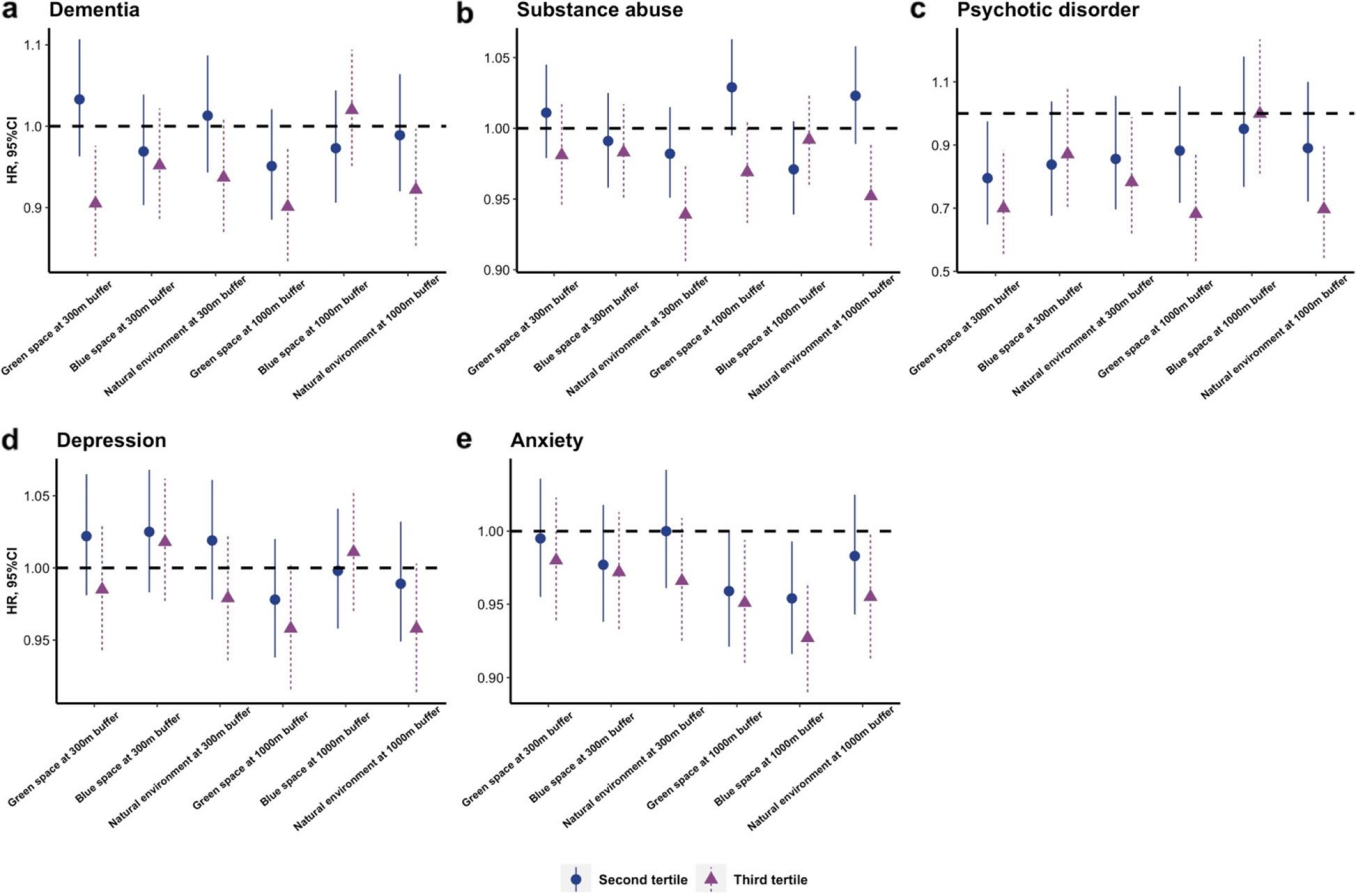
The independent associations of green and blue space and natural environment with specific psychiatric disorders (HR, hazard ratio; CI, confidence interval; the estimates were adjusted for age, sex, ethnicity, socioeconomic status; BMI, household income, education group, smoking status, alcohol drinker status, and physical activity, and the references were the first tertile of green and blue space and nature environment, respectively)

The strength of the associations varied across psychiatric disorders (Fig. 1 and Additional file 1: Table S3) with the strongest association for a protective effect of GBN exposure observed for psychotic disorders: the third tertile of both green space at 300 m (HR:0.700, 95% CI: 0.555–0.884) and 1000 m buffer (HR:0.682, 95% CI: 0.532–0.874) was associated with an approximate 30% risk of psychotic disorders and approximately 20% and 30% for the natural environment at 300 m (HR:0.783, 95% CI: 0.620–0.988) and 1000 m buffer (HR:0.697, 95% CI: 0.542–0.896), respectively. Compared with the first tertile of exposure, the risk of incident dementia decreased with exposure to the third tertile of greenspace at 300 m (HR:0.905, 95% CI: 0.840–0.976) and 1000 m buffer (HR:0.901, 95% CI: 0.834–0.973) and with the natural environment at 1000 m buffer (HR:0.922, 95% CI: 0.853–0.997). The natural environment at 300 m (HR:0.939, 95% CI: 0.906–0.974) and 1000 m buffer (HR:0.952, 95% CI: 0.917–0.989) were statistically associated with an increased risk of substance abuse. There was a reduction in the risk of incident anxiety among those exposed to the third tertile of greenspace (HR:0.951, 95% CI: 0.910–0.994) and the natural environment (HR:0.955, 95% CI: 0.913–0.999) at 1000 m buffer and with the second and third tertile of blue space at 1000 m buffer. We did not observe a significant effect of GBN on incident depression. Using an ordinal rather than a categorical scale of GBN by tertiles did not materially influence the results.

**Fig. 2:**
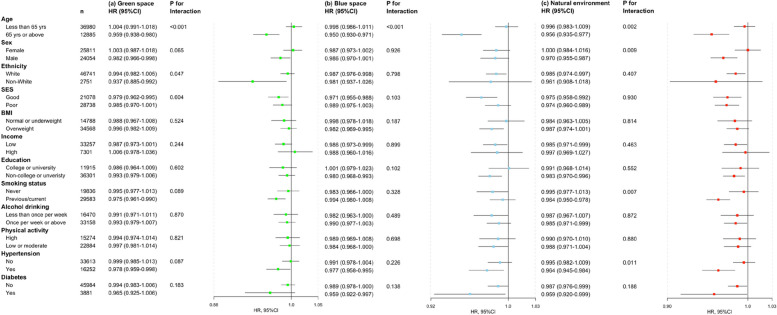
The associations of green and blue space and natural environment at 300 m buffer with any psychiatric disorder by stratified factors (HR, hazard ratio; CI, confidence interval; SES, socioeconomic status; BMI: body mass index, the estimates were adjusted for ethnicity, SES, BMI, household income, education group, smoking status, alcohol drinker status, and physical activity, and relative factors were not included in the models when performing corresponding subgroup analysis)

**Fig. 3:**
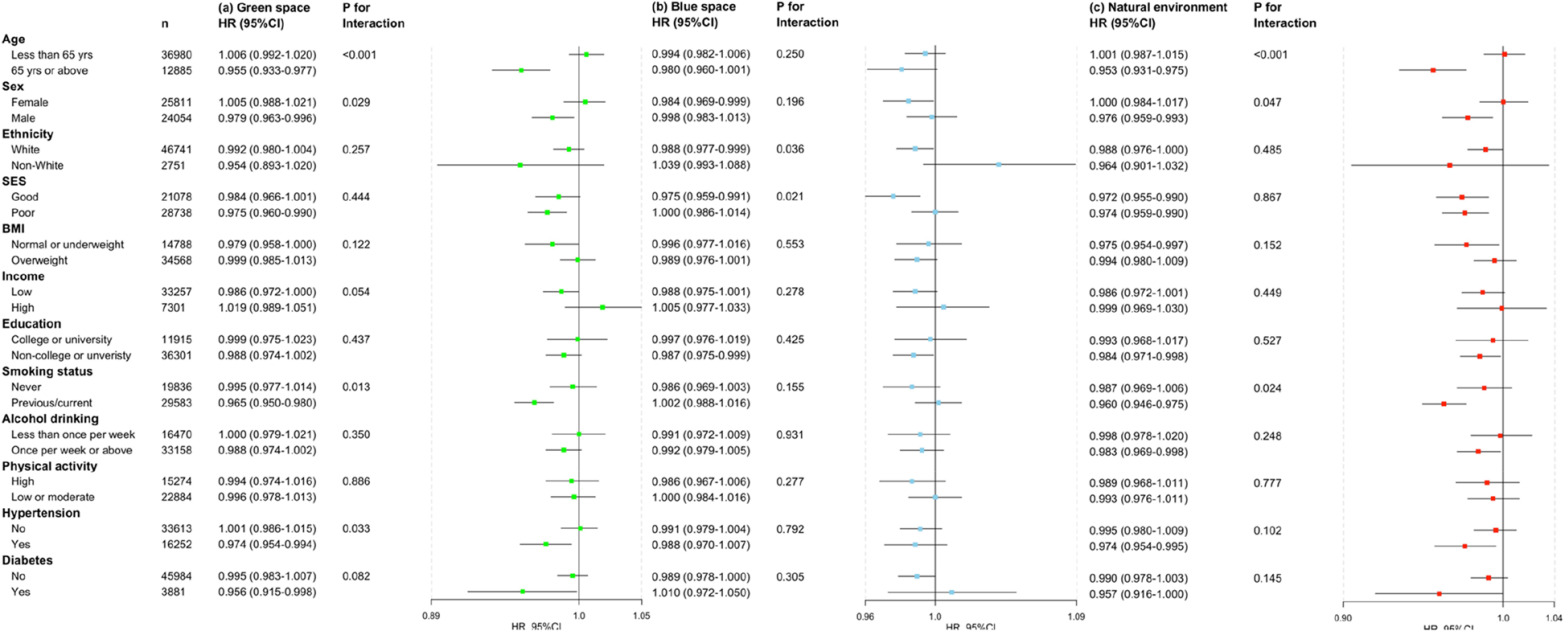
The associations of green and blue space and natural environment at 1000 m buffer with any psychiatric disorder by stratified factors (HR, hazard ratio; CI, confidence interval; SES, socioeconomic status; BMI: body mass index, the estimates were adjusted for ethnicity, SES, BMI, household income, education group, smoking status, alcohol drinker status, and physical activity, and relative factors were not included in the models when performing corresponding subgroup analysis)

Stratified analyses indicated that the protective associations of green space at 300 m and 1000 m buffer, blue space at 300 m buffer, and the natural environment at 300 m and at 1000 m buffer with psychiatric disorders were stronger among those aged ≥ 65 years compared with younger individuals (Figs. 2 and 3). There was also some evidence to indicate that the effects of green space and the natural environment were stronger in men than in women. Similarly, stronger effects of GBN on incident psychiatric disorders were observed among those individuals with a history of cigarette smoking and those with hypertension and type 2 diabetes.

Results of the sensitivity analyses indicated that the associations between GBN and any psychiatric disorder did not materially change after adjusting for PM_10_, noise pollution, time spent outdoors, and other variables (Additional file 1: Table S4-S6) and using different cut-offs of GBN (Additional file 1: Table S7).

## Discussion

This is the largest longitudinal study to explore the prospective associations of GBN with any or specific psychiatric disorders in middle-aged and older adults. Overall, there was evidence of a weak protective and independent effect of exposure to GBN on risk of psychiatric disorders, with the strongest association observed with psychotic disorders. The relationship was robust after adjusting for the potential confounding effect of, among other factors, noise and air pollution. In contrast, there was no evidence that exposure to GBN was associated with incident depression. The protective effect of GBN on the independent risk of incident psychiatric disorders was stronger for specific population subgroups, namely those aged ≥ 65 years, men, and those with pre-existing comorbidities.

A previous cross-sectional study from China had indicated that living near greenspace was negatively associated with psychiatric symptoms [42], whereas studies from Europe and the USA reported no significant association for questionnaire-based psychiatric symptoms [43–45] or diagnoses of psychiatric disorders [16, 46]. Similar with certain studies, our analysis also showed no beneficial effect of higher greenspace coverage on risk of any psychiatric disorder consistent with previous studies. Specifically, previous observational studies reported that greenspace was associated with a lower risk of depression or depressive symptoms [9–13, 47]. However, the findings from our current study, as well as others [17, 18], did not support a relationship between greenspace and risk of depression. These disparities in findings may be due to differences in study design and how a diagnosis of a psychiatric condition was made [10–13]. Between study differences in buffer size or index related to greenspace may also have contributed to the lack of consistency in study findings [9, 11, 47]. In line with most previous studies, this study showed a beneficial effect of green space on incident anxiety [11, 14] and dementia [7, 8]. To our knowledge, this is the first study to report on a possible protective effect of greater exposure to greenspace coverage on subsequent risk of psychotic disorders, for example, schizophrenia, schizotypal disorders, or schizoaffective disorders. Further studies are warranted to validate these findings in different populations and to understand the potential mechanistic pathways that may underpin the association.

In line with previous evidence from observational studies [16, 19, 48, 49], this study also showed that blue space coverage was statistically associated with decreased risk of any psychiatric disorder consistent with findings from a systematic review [50]. Although a few studies have reported the benefits of blue space coverage with specific psychiatric disorders, for example depression [12, 13, 18] and anxiety [12, 14, 19], the findings were controversial and had visible heterogeneity. We examined the associations of blue space with five specific psychiatric disorders and only detected significant associations of blue space at 1000 m buffer with incident anxiety. Nonetheless, more experimental studies are needed to confirm these associations.

Evidence from longitudinal studies with long-term follow-up regarding the potential relationship that the total natural environment may have with psychiatric disorders is limited. Findings from our study suggested that the natural environment within a 1000-m buffer could lower an individuals’ susceptibility to developing a psychiatric disorder. A recent meta-analysis and systematic review involving 33 studies reported that short-term exposure to the natural environment was associated with a small protective effect on depressive mood [20]. Findings from our study were in agreement with this review such that exposure to the natural environment at 1000 m buffer was mildly protective against incident depression (HR = 0.955 for the third tertile). Furthermore, Zhang et al. performed a systematic review and found that exposure to the natural environment could alleviate anxiety [21], which is in agreement with results from our study.

Epidemiological data have consistently demonstrated that GBN is related to decreased risk of chronic disease [3–6, 51]. The physiological and behavioral mechanisms underpinning this relationship may also have a mediating role in the association between GBN and risk of psychiatric disorders: greater exposure to GBN might be promote less sedentary behavior and more physical activity, which could subsequently improve mental health [52]. Additionally, a study from China reported that lower green space was associated with lower sleep quality, which is itself a risk factor for psychiatric disorders [53].

Our current study found that the protective effect of GBN on psychiatric disorders was stronger among those aged ≥ 65 years compared with younger individuals. This effect from age may reflect the retirement status of older individuals (especially men) who as a result would be spending more time at home than those still in the paid workforce. Alternatively, older individuals are likely to have more complex comorbid conditions (e.g., hypertension and diabetes) which may mediate the link between exposure to GBN and risk of psychiatric disorders. Our findings that men, in particular, may benefit more from exposure to GBN compared with women are congruent with previous studies from the UKB. In those studies, the beneficial effect of exposure to green or/and blue space on inflammatory bowel diseases [6] and cardiovascular disease and respiratory disease mortality rates [54] was stronger in men than in women. These sex differences may reflect the higher prevalence of more suboptimal lifestyle behaviors in men compared with women, e.g., current smoking, alcohol consumption, poor diet, and low levels of physical activity [55]. Although we adjusted for these and other potential confounders, residual confounding is likely to have remained. It should be noted however that when we stratified by level of physical activity, the results were not materially different. Rather than relying on self-report measures of physical activity, future studies that include device-measured physical activity are likely to be more informative. In particular, using wearable devices to track activity levels would enable adjustment for the amount and intensity of physical activity while exposed to GBN, something which we were unable to address in the current study.

### Strengths and limitations

Several strengths of this study should be mentioned. First, the UKB is a large-scale well-characterized, population-based cohort study with information on many variables. This enables us to adjust for potential confounders of the relationship between exposure to GBN with psychiatric disorders. We were also able to undertake sensitivity analyses, for example, those involving noise and air pollution data. Although the effect is relatively small, the findings in the current study are robust and reliable. Second, the long duration of follow-up in UKB is unique and enabled us to look at the long-term effects of GBN on psychiatric disorders. Finally, the current study not only reported on the prospective associations with any psychiatric disorder but examined important specific psychiatric disorders.

Some limitations should also be mentioned when explaining the results of this study. First, we only captured the data of GBN at 300 m and 1000 m buffer; more detailed data at a buffer of 100 m were not available. Moreover, urbanicity, which is a potential confounder for the association between GBN and psychiatric disorders, was lacked update to the study period in UKB and hence could not be adjusted for in the analysis. Furthermore, although sensitivity analyses that had adjusted for outdoor time showed no material impact on the main findings, it is important to mention that there may have been information bias, namely misclassification bias, when dividing the participants into different groups of exposure to GBN, especially for the uncertain physical or/and visual accessibility of GBN and how much exposure to GBN spaces individuals received. Additionally, exposures were only collected at baseline (2010), which may have led to misclassification if an individual changed residential location or factors relating to socioeconomic status or lifestyle behaviors (e.g., changing physical activity levels). Although we included a wide range of potential confounders, residual confounding by unmeasured factors such as social support, job-related stress, or other neighborhood-level variables is likely to have remained. Finally, UKB had a low rate (5%) of recruitment and a limited age group from 37 to 73 years [22], which may have introduced some selection bias and therefore limits the generalizability of the conclusions to the general UK population.

## Conclusions

In summary, greater exposure to GBN was associated with decreased risk for any or specific psychiatric disorders in middle-aged and older adults. There was evidence that the effects may be greater among older individuals, men, and those with pre-existing conditions. Further studies are warranted to investigate the social, biological, and physiological interplay more fully between the environment and an individuals’ mental health.

### Supplementary Information

**Additional file 1: Fig. S1.** Study flow diagram. **Table S1.** The definition of psychiatric disorders in this study. **Table S2.** Data field and definition of the included covariates. **Table S3.** The independent associations of green and blue space with specific psychiatric disorders. **Table S4.** Sensitivity analysis for the associations of green, blue space and natural environment with any psychiatric disorder by adjusting air and noise pollution. **Table S5.** Sensitivity analysis for the associations of green, blue space and natural environment with any psychiatric disorder by adjusting additional factors. **Table S6.** Sensitivity analysis for the associations of green, blue space and natural environment with any psychiatric disorder by omitting the participants with missing values and any psychiatric disorder for the first two years. **Table S7.** Sensitivity analysis for the associations of green, blue space and natural environment with any psychiatric disorder by using different cut-offs.

## Data Availability

UK Biobank data could be obtained on application from https://www.ukbiobank.ac.uk/enable-your-research/apply-for-access.

## Abbreviations

GBN: Green space, blue space, and the natural environment
HR: Hazard ratio
CI: Confidence interval
DALYs: Disability-adjusted life-years
UKB: UK Biobank
GLUD: Generalized Land Use Database
CEH: Ecology and Hydrology
LCM: Land Cover Map
ICD: International Classification of Disease
SES: Socioeconomic status
BMI: Body mass index
T2D: Type 2 diabetes
MET: Metabolic Equivalent Task
IPAQ: International Physical Activity Questionnaire
MICE: Multivariate imputations by chained equations
PM: Particular matter
ESCAPE: European Study of Cohorts for Air Pollution Effects
GP: General practitioner
