# Supplementary material for: Exposure to residential green and blue space and the natural environment is associated with a lower incidence of psychiatric disorders in middle-aged and older adults: findings from the UK Biobank
Source: BMC Med. 2024 Jan 15;22:15. doi: 10.1186/s12916-023-03239-1 (PMC10789017; doi:10.1186/s12916-023-03239-1)
Supplement: Supplementary file 1 — Additional file 1: Fig. S1. Study flow diagram. Table S1. The definition of psychiatric disorders in this study. Table S2. Data field and definition of the included covariates. Table S3. The independent associations of green and blue space with specific psychiatric disorders. Table S4. Sensitivity analysis for the associations of green, blue space and natural environment with any psychiatric disorder by adjusting air and noise pollution. Table S5. Sensitivity analysis for the associations of green, blue space and natural environment with any psychiatric disorder by adjusting additional factors. Table S6. Sensitivity analysis for the associations of green, blue space and natural environment with any psychiatric disorder by omitting the participants with missing values and any psychiatric disorder for the first two years. Table S7. Sensitivity analysis for the associations of green, blue space and natural environment with any psychiatric disorder by using different cut-offs. [file 12916_2023_3239_MOESM1_ESM.docx]

**Additional file 1**

**Fig. S1:** Study flow diagram

**Table S1:** The definition of psychiatric disorders in this study

**Table S2:** Data field and definition of the included covariates

**Table S3:** The independent associations of green and blue space with specific psychiatric disorders

**Table S4:** Sensitivity analysis for the associations of green, blue space and natural environment with any psychiatric disorder by adjusting air and noise pollution

**Table S5:** Sensitivity analysis for the associations of green, blue space and natural environment with any psychiatric disorder by adjusting additional factors

**Table S6:** Sensitivity analysis for the associations of green, blue space and natural environment with any psychiatric disorder by omitting the participants with missing values and any psychiatric disorder for the first two years

**Table S7:** Sensitivity analysis for the associations of green, blue space and natural environment with any psychiatric disorder by using different cut-offs

**Fig. S1: Study flow diagram**

Participants in the present UK biobank dataset (n=502411)

Excluded participants lost to follow up or withdraw (n=1320)

Participants could be followed up (n=501091)

Excluded participants who had missing value for green space, blue space, and natural environment (n=61388)

Participants included for analyses (n=363047)

Excluded participants who had at least one psychiatric disorder at baseline (n=76656)

**Table S1:** The definition of psychiatric disorders in this study

| **Psychiatric disorder** | **ICD-10 code** | **Field ID in UK biobank for occurrence and time** |
| --- | --- | --- |
|  |  |  |
| Any psychiatric disorder | F00-F99 | 130836-130991 |
| Dementia | F00-F03 | 130836-130843 |
| Substance misuse | F10-F19 | 130854-130873 |
| Psychotic disorder | F20-F29 | 130874-130889 |
| Depression | F32-F33 | 130894-130897 |
| Anxiety | F40-F41 | 130904-130907 |

**Table S2:** Data field and definition of the included covariates

| **Covariates** | **Data field** | **Question and description** | **Type of variables** |
| --- | --- | --- | --- |
| Age | 21002 | Age at first recruitment | Continuous |
| Sex | 31 | Self-reported sex during the initial Assessment Centre visit | Categorical (female and male) |
| Ethnicity | 21000 | Self-reported ethnic background during the initial Assessment Centre visit | Categorical (White or others) |
| Socioeconomic status | 189 | Townsend deprivation index, which could reflect socioeconomic status, was calculated immediately prior to participant joining UK Biobank. Based on the preceding national census output areas. Each participant is assigned a score corresponding to the output area in which their postcode is located. A higher score means greater socioeconomic deprivation. | Categorical (quartiles) |
| BMI | 21001 | BMI value here is constructed from height and weight measured during the initial Assessment Centre visit. | Categorical (underweight/normal weight, overweight or obese) |
| Household income before tax per year (£) | 738 | Touchscreen question "What is the average total income before tax received by your HOUSEHOLD per year?" | Categorical (<£18000, £18000-£30999, £31000-£51999, £52000-£100000, or >£100000) |
| Education group | 6138 | Touchscreen question "Which of the following qualifications do you have? | Categorical (college or university degree; any school degree including A-level, AS-level, O-level, GCSE, CSE; vocational qualification (NVQ, HND, or HNC) or other professional qualifications; or none of the above) |
| Smoking status | 20116 | Directly use the variable 20116, which is a summary for many questions about smoking | Categorical (never, previous, or current) |
| Alcohol drinker status | 1558 | Touchscreen question "About how often do you drink alcohol?" | Categorical (daily or almost daily, above one time per week, one to three times a month, Special occasions only, never) |
| Physical activity | 22032 | This category contains derived MET (Metabolic Equivalent Task) scores data based on IPAQ (International Physical Activity Questionnaire) guidelines. | Categorical (low, moderate or high) |
| Hypertension | 131294,  131295 | The diagnoses and time of occurrence were obtained from “first occurrence fields” provided by UKB (data category: 2409) and coded by International Classification of Disease, 10^th^ version (ICD-10), which included data from primary care, hospital inpatient record, self-reported medical condition, and death registers. | Yes or no |
| Type 2 diabetes | 130708,  130709,  130714,  130715 |  | Yes or no |


**Table S3: The independent associations of green and blue space with specific psychiatric disorders**

| **Exposure** | **HR (95%CI)** # | | | | |
| --- | --- | --- | --- | --- | --- |
|  | **dementia** | **Substance misuse** | **Psychotic disorder** | **Depression** | **Anxiety** |
| **Green space, 300m buffer** |  |  |  |  |  |
| First tertile (the lowest) | Reference | Reference | Reference | Reference | Reference |
| Second tertile | 1.033 (0.963-1.107) | 1.011 (0.979-1.045) | 0.795 (0.648-0.975) * | 1.022 (0.981-1.065) | 0.995 (0.955-1.036) |
| Third tertile (the highest) | 0.905 (0.840-0.976) ** | 0.981 (0.946-1.017) | 0.700 (0.555-0.884) ** | 0.986 (0.943-1.030) | 0.980 (0.939-1.023) |
| Ordinal scale | 0.953 (0.918-0.988) * | 0.992 (0.974-1.009) | 0.832 (0.741-0.934) ** | 0.993 (0.972-1.015) | 0.990 (0.969-1.011) |
| **Blue space, 300m buffer** |  |  |  |  |  |
| First tertile (the lowest) | Reference | Reference | Reference | Reference | Reference |
| Second tertile | 0.969 (0.903-1.039) | 0.991 (0.958-1.025) | 0.838 (0.676-1.038) | 1.025 (0.983-1.068) | 0.977 (0.938-1.018) |
| Third tertile (the highest) | 0.952 (0.886-1.022) | 0.983 (0.951-1.017) | 0.871 (0.704-1.077) | 1.018 (0.976-1.062) | 0.972 (0.933-1.013) |
| Ordinal scale | 0.975 (0.941-1.011) | 0.992 (0.975-1.008) | 0.930 (0.835-1.036) | 1.009 (0.988-1.030) | 0.986 (0.966-1.006) |
| **Natural environment, 300m buffer** |  | 30. |  |  |  |
| First tertile (the lowest) | Reference | Reference | Reference | Reference | Reference |
| Second tertile | 1.013 (0.943-1.087) | 0.982 (0.951-1.015) | 0.856 (0.696-1.055) | 1.019 (0.978-1.061) | 1.000 (0.961-1.042) |
| Third tertile (the highest) | 0.937 (0.870-1.010) | 0.939 (0.906-0.974) *** | 0.783 (0.620-0.988) * | 0.979 (0.936-1.023) | 0.966 (0.925-1.009) |
| Ordinal scale | 0.968 (0.933-1.005) | 0.970 (0.953-0.988) *** | 0.882 (0.786-0.991) * | 0.990 (0.968-1.012) | 0.983 (0.962-1.004) |
| **Green space, 1000m buffer** |  |  |  |  |  |
| First tertile (the lowest) | Reference | Reference | Reference | Reference | Reference |
| Second tertile | 0.951 (0.885-1.021) | 1.029 (0.995-1.063) | 0.882 (0.717-1.086) | 0.978 (0.938-1.020) | 0.959 (0.921-1.000) * |
| Third tertile (the highest) | 0.901 (0.834-0.973) ** | 0.969 (0.933-1.006) | 0.682 (0.532-0.874) ** | 0.958 (0.916-1.003) | 0.951 (0.910-0.994) * |
| Ordinal scale | 0.949 (0.913-0.986) ** | 0.987 (0.969-1.006) | 0.831 (0.736-0.939) ** | 0.979 (0.957-1.001) | 0.975 (0.954-0.997) * |
| **Blue space, 1000m buffer** |  |  |  |  |  |
| First tertile (the lowest) | Reference | Reference | Reference | Reference | Reference |
| Second tertile | 0.973 (0.906-1.044) | 0.971 (0.939-1.005) | 0.951 (0.767-1.180) | 0.998 (0.958-1.041) | 0.954 (0.916-0.993) * |
| Third tertile (the highest) | 1.020 (0.951-1.094) | 0.992 (0.960-1.025) | 0.999 (0.809-1.235) | 1.011 (0.970-1.054) | 0.927 (0.890-0.965) *** |
| Ordinal scale | 1.010 (0.975-1.046) | 0.996 (0.980-1.013) | 1.000 (0.899-1.112) | 1.006 (0.985-1.026) | 0.963 (0.943-0.982) *** |
| **Natural environment, 1000m buffer** |  |  |  |  |  |
| First tertile (the lowest) | Reference | Reference | Reference | Reference | Reference |
| Second tertile | 0.989 (0.920-1.064) | 1.023 (0.989-1.058) | 0.890 (0.721-1.100) | 0.989 (0.949-1.032) | 0.983 (0.943-1.025) |
| Third tertile (the highest) | 0.922 (0.853-0.997) * | 0.952 (0.917-0.989) * | 0.697 (0.542-0.896) ** | 0.958 (0.914-1.003) | 0.955 (0.913-0.999) * |
| Ordinal scale | 0.960 (0.923-0.998) * | 0.979 (0.960-0.997) * | 0.840 (0.743-0.950) ** | 0.979 (0.957-1.002) | 0.977 (0.956-0.999) * |

Abbreviation: HR, hazard ratio; CI, confidence interval.

# *p*<0.001, ****** *p*<0.01, **p*<0.05. The models were adjusted for age, sex, ethnicity, socioeconomic status, BMI, household income, education group, smoking status, alcohol drinker status, and physical activity.

**Table S4: Sensitivity analysis for the associations of green, blue space and natural environment with any psychiatric disorder by adjusting air and noise pollution**

| **Exposure** | **HR (95%CI)** # | | |
| --- | --- | --- | --- |
|  | **Original estimates** | **Adjusted by PM_10_** | **Adjusted by noise** |
| **Green space, 300m buffer** |  |  |  |
| First tertile (the lowest) | Reference | Reference | Reference |
| Second tertile | 1.010 (0.988-1.031) | 1.009 (0.988-1.031) | 1.010 (0.989-1.032) |
| Third tertile (the highest) | 0.984 (0.961-1.006) | 0.981 (0.958-1.004) | 0.983 (0.961-1.006) |
| Ordinal scale | 0.992 (0.981-1.004) | 0.991 (0.980-1.003) | 0.992 (0.981-1.004) |
| **Blue space, 300m buffer** |  |  |  |
| First tertile (the lowest) | Reference | Reference | Reference |
| Second tertile | 0.983 (0.961-1.004) | 0.982 (0.961-1.004) | 0.982 (0.961-1.004) |
| Third tertile (the highest) | 0.973 (0.952-0.994) * | 0.973 (0.952-0.994) * | 0.973 (0.952-0.994) * |
| Ordinal scale | 0.986 (0.976-0.997) * | 0.986 (0.975-0.997) * | 0.986 (0.976-0.997) * |
| **Natural environment, 300m buffer** |  |  |  |
| First tertile (the lowest) | Reference | Reference | Reference |
| Second tertile | 1.003 (0.982-1.025) | 1.002 (0.981-1.024) | 1.003 (0.982-1.025) |
| Third tertile (the highest) | 0.970 (0.948-0.992) ** | 0.965 (0.943-0.989) ** | 0.970 (0.948-0.992) ** |
| Ordinal scale | 0.985 (0.974-0.997) ** | 0.983 (0.972-0.995) ** | 0.985 (0.974-0.997) ** |
| **Green space, 1000m buffer** |  |  |  |
| First tertile (the lowest) | Reference | Reference | Reference |
| Second tertile | 1.013 (0.991-1.035) | 1.013 (0.991-1.035) | 1.013 (0.991-1.036) |
| Third tertile (the highest) | 0.983 (0.960-1.007) | 0.981 (0.957-1.005) | 0.983 (0.960-1.007) |
| Ordinal scale | 0.992 (0.980-1.004) | 0.991 (0.979-1.003) | 0.992 (0.980-1.004) |
| **Blue space, 1000m buffer** |  |  |  |
| First tertile (the lowest) | Reference | Reference | Reference |
| Second tertile | 0.974 (0.953-0.995) * | 0.974 (0.953-0.995) * | 0.974 (0.953-0.995) * |
| Third tertile (the highest) | 0.981 (0.960-1.002) | 0.981 (0.960-1.002) | 0.981 (0.960-1.002) |
| Ordinal scale | 0.990 (0.980-1.001) | 0.990 (0.980-1.001) | 0.990 (0.980-1.001) |
| **Natural environment, 1000m buffer** |  |  |  |
| First tertile (the lowest) | Reference | Reference | Reference |
| Second tertile | 1.014 (0.992-1.036) | 1.013 (0.991-1.035) | 1.014 (0.992-1.036) |
| Third tertile (the highest) | 0.975 (0.952-0.999) * | 0.972 (0.948-0.996) * | 0.975 (0.952-0.999) * |
| Ordinal scale | 0.988 (0.976-1.000) * | 0.987 (0.975-0.999) * | 0.988 (0.976-1.000) * |

HR: hazard ratio, CI: confidence interval.

**p*<0.05, ***p*<0.01, ****p*<0.001

# The estimates were adjusted for age, sex, ethnicity, socioeconomic status, BMI, household income, education group, smoking status, alcohol drinker status, and physical activity.

**Table S5: Sensitivity analysis for the associations of green, blue space and natural environment with any psychiatric disorder by adjusting additional factors**

| **Exposure** | **HR (95%CI)** # | | |
| --- | --- | --- | --- |
|  | **Original estimates** | **Adjusted by outdoor time** | **Adjusted by** **history of consulting from a psychiatrist or GP** |
| **Green space, 300m buffer** |  |  |  |
| First tertile (the lowest) | Reference | Reference | Reference |
| Second tertile | 1.010 (0.988-1.031) | 1.009 (0.988-1.031) | 1.011 (0.990-1.033) |
| Third tertile (the highest) | 0.984 (0.961-1.006) | 0.982 (0.960-1.005) | 0.987 (0.965-1.010) |
| Ordinal scale | 0.992 (0.981-1.004) | 0.991 (0.980-1.003) | 0.994 (0.983-1.005) |
| **Blue space, 300m buffer** |  |  |  |
| First tertile (the lowest) | Reference | Reference | Reference |
| Second tertile | 0.983 (0.961-1.004) | 0.982 (0.961-1.003) | 0.984 (0.963-1.005) |
| Third tertile (the highest) | 0.973 (0.952-0.994) * | 0.973 (0.952-0.994) * | 0.974 (0.953-0.995) * |
| Ordinal scale | 0.986 (0.976-0.997) * | 0.987 (0.976-0.998) * | 0.987 (0.976-0.997) * |
| **Natural environment, 300m buffer** |  |  |  |
| First tertile (the lowest) | Reference | Reference | Reference |
| Second tertile | 1.003 (0.982-1.025) | 1.002 (0.981-1.024) | 1.005 (0.983-1.027) |
| Third tertile (the highest) | 0.970 (0.948-0.992) ** | 0.968 (0.946-0.991) ** | 0.974 (0.952-0.996) * |
| Ordinal scale | 0.985 (0.974-0.997) ** | 0.984 (0.973-0.996) ** | 0.987 (0.976-0.999) * |
| **Green space, 1000m buffer** |  |  |  |
| First tertile (the lowest) | Reference | Reference | Reference |
| Second tertile | 1.013 (0.991-1.035) | 1.013 (0.991-1.035) | 1.016 (0.994-1.038) |
| Third tertile (the highest) | 0.983 (0.960-1.007) | 0.981 (0.958-1.005) | 0.988 (0.965-1.011) |
| Ordinal scale | 0.992 (0.980-1.004) | 0.991 (0.979-1.003) | 0.994 (0.983-1.006) |
| **Blue space, 1000m buffer** |  |  |  |
| First tertile (the lowest) | Reference | Reference | Reference |
| Second tertile | 0.974 (0.953-0.995) * | 0.974 (0.953-0.996) * | 0.974 (0.953-0.995) * |
| Third tertile (the highest) | 0.981 (0.960-1.002) | 0.981 (0.960-1.002) | 0.981 (0.961-1.003) |
| Ordinal scale | 0.990 (0.980-1.001) | 0.991 (0.980-1.001) | 0.991 (0.980-1.001) |
| **Natural environment, 1000m buffer** |  |  |  |
| First tertile (the lowest) | Reference | Reference | Reference |
| Second tertile | 1.014 (0.992-1.036) | 1.013 (0.991-1.036) | 1.014 (0.992-1.037) |
| Third tertile (the highest) | 0.975 (0.952-0.999) * | 0.973 (0.950-0.997) * | 0.980 (0.957-1.004) |
| Ordinal scale | 0.988 (0.976-1.000) * | 0.987 (0.975-0.999) * | 0.990 (0.979-1.002) |

HR: hazard ratio, CI: confidence interval, GP: general practitioner.

**p*<0.05, ***p*<0.01, ****p*<0.001

# The estimates were adjusted for age, sex, ethnicity, socioeconomic status, BMI, household income, education group, smoking status, alcohol drinker status, and physical activity.

**Table S6: Sensitivity analysis for the associations of green, blue space and natural environment with any psychiatric disorder by omitting the participants with missing values and any psychiatric disorder for the first two years**

| **Exposure** | **HR (95%CI)** # | | |
| --- | --- | --- | --- |
|  | **Original estimates** | **Without missing values for all included variables** | **Excluding any psychiatric disorder for the first two years after baseline** |
| **Green space, 300m buffer** |  |  |  |
| First tertile (the lowest) | Reference | Reference | Reference |
| Second tertile | 1.010 (0.988-1.031) | 1.007 (0.981-1.035) | 1.006 (0.983-1.029) |
| Third tertile (the highest) | 0.984 (0.961-1.006) | 0.987 (0.960-1.015) | 0.971 (0.947-0.995) * |
| Ordinal scale | 0.992 (0.981-1.004) | 0.994 (0.980-1.008) | 0.986 (0.974-0.998) * |
| **Blue space, 300m buffer** |  |  |  |
| First tertile (the lowest) | Reference | Reference | Reference |
| Second tertile | 0.983 (0.961-1.004) | 0.979 (0.953-1.006) | 0.979 (0.956-1.001) |
| Third tertile (the highest) | 0.973 (0.952-0.994) * | 0.970 (0.945-0.997) * | 0.975 (0.953-0.998) * |
| Ordinal scale | 0.986 (0.976-0.997) * | 0.985 (0.972-0.998) * | 0.988 (0.976-0.999) * |
| **Natural environment, 300m buffer** |  |  |  |
| First tertile (the lowest) | Reference | Reference | Reference |
| Second tertile | 1.003 (0.982-1.025) | 1.003 (0.977-1.031) | 0.988 (0.965-1.010) |
| Third tertile (the highest) | 0.970 (0.948-0.992) ** | 0.983 (0.955-1.011) | 0.952 (0.929-0.976) *** |
| Ordinal scale | 0.985 (0.974-0.997) ** | 0.992 (0.978-1.006) | 0.976 (0.964-0.988) *** |
| **Green space, 1000m buffer** |  |  |  |
| First tertile (the lowest) | Reference | Reference | Reference |
| Second tertile | 1.013 (0.991-1.035) | 1.026 (0.999-1.055) | 0.999 (0.976-1.022) |
| Third tertile (the highest) | 0.983 (0.960-1.007) | 0.994 (0.966-1.024) | 0.962 (0.938-0.987) ** |
| Ordinal scale | 0.992 (0.980-1.004) | 0.997 (0.983-1.012) | 0.981 (0.969-0.994) ** |
| **Blue space, 1000m buffer** |  |  |  |
| First tertile (the lowest) | Reference | Reference | Reference |
| Second tertile | 0.974 (0.953-0.995) * | 0.971 (0.946-0.998) * | 0.978 (0.956-1.001) |
| Third tertile (the highest) | 0.981 (0.960-1.002) | 0.986 (0.961-1.013) | 0.988 (0.965-1.011) |
| Ordinal scale | 0.990 (0.980-1.001) | 0.993 (0.980-1.007) | 0.994 (0.983-1.005) |
| **Natural environment, 1000m buffer** |  |  |  |
| First tertile (the lowest) | Reference | Reference | Reference |
| Second tertile | 1.014 (0.992-1.036) | 1.027 (0.999-1.055) | 0.992 (0.969-1.016) |
| Third tertile (the highest) | 0.975 (0.952-0.999) * | 0.988 (0.960-1.018) | 0.953 (0.929-0.978) *** |
| Ordinal scale | 0.988 (0.976-1.000) * | 0.994 (0.980-1.009) | 0.977 (0.964-0.989) *** |

HR: hazard ratio, CI: confidence interval, GP: general practitioner. **p*<0.05, ***p*<0.01, ****p*<0.001

# The estimates were adjusted for age, sex, ethnicity, socioeconomic status, BMI, household income, education group, smoking status, alcohol drinker status, and physical activity.

**Table S7: Sensitivity analysis for the associations of green, blue space and natural environment with any psychiatric disorder by using different cut-offs**

| **Exposure** | **HR (95%CI)** # | | | | | |
| --- | --- | --- | --- | --- | --- | --- |
|  | **Green space, 300m buffer** | **Blue space, 300m buffer** | **Natural environment, 300m buffer** | **Green space, 1000m buffer** | **Blue space, 1000m buffer** | **Natural environment, 1000m buffer** |
| **Three groups (original estimates)** |  |  |  |  |  |  |
| First tertile (the lowest) | Reference | Reference | Reference | Reference | Reference | Reference |
| Second tertile | 1.010 (0.988-1.031) | 0.983 (0.961-1.004) | 1.003 (0.982-1.025) | 1.013 (0.991-1.035) | 0.974 (0.953-0.995) * | 1.014 (0.992-1.036) |
| Third tertile (the highest) | 0.984 (0.961-1.006) | 0.973 (0.952-0.994) * | 0.970 (0.948-0.992) ** | 0.983 (0.960-1.007) | 0.981 (0.960-1.002) | 0.975 (0.952-0.999) * |
| **Three groups** |  |  |  |  |  |  |
| $\leq$ 20 percentiles | Reference | Reference | Reference | Reference | Reference | Reference |
| > 20 to 80 percentiles | 1.020 (0.997-1.044) | 0.988 (0.966-1.011) | 1.017 (0.995-1.041) | 1.033 (1.010-1.058) ** | 0.989 (0.967-1.012) | 1.021 (0.997-1.045) |
| > 80 percentiles | 0.968 (0.939-0.998) * | 0.963 (0.937-0.990) ** | 0.949 (0.921-0.978) *** | 0.976 (0.947-1.007) | 0.987 (0.960-1.014) | 0.967 (0.938-0.998) * |
| **Two groups** |  |  |  |  |  |  |
| $\leq$ 50 percentiles | Reference | Reference | Reference | Reference | Reference | Reference |
| > 50 percentiles | 1.002 (0.984-1.021) | 0.977 (0.960-0.994) ** | 0.984 (0.966-1.003) | 0.984 (0.965-1.002) | 0.975 (0.958-0.992) ** | 0.970 (0.952-0.989) ** |
| **Four groups** |  |  |  |  |  |  |
| $\leq$ 25 percentiles | Reference | Reference | Reference | Reference | Reference | Reference |
| > 25 to 50 percentiles | 1.000 (0.976-1.025) | 0.999 (0.975-1.024) | 1.015 (0.991-1.041) | 1.029 (1.004-1.054) * | 1.004 (0.979-1.029) | 1.028 (1.002-1.053) * |
| > 50 to 75 percentiles | 1.028 (1.002-1.054) | 0.988 (0.964-1.013) | 1.014 (0.989-1.040) | 1.011 (0.985-1.038) | 0.961 (0.938-0.986) ** | 0.996 (0..971-1.023) |
| > 75 percentiles | 0.971 (0.945-0.997) * | 0.965 (0.42-0.989) ** | 0.966 (0.941-0.992) * | 0.981 (0.954-1.008) | 0.993 (0.968-2.017) | 0.968 (0.941-0.995) * |

HR: hazard ratio, CI: confidence interval, GP: general practitioner. **p*<0.05, ***p*<0.01, ****p*<0.001

# The estimates were adjusted for age, sex, ethnicity, socioeconomic status, BMI, household income, education group, smoking status, alcohol drinker status, and physical activity.
